# Prior antiretroviral therapy exposure among clients presenting for HIV treatment initiation in South Africa: an exploratory mixed-methods study using multiple indicators of exposure

**DOI:** 10.1186/s12879-025-11340-4

**Published:** 2025-07-26

**Authors:** Mariet Benade, Mhairi Maskew, Vinolia Ntjikelane, Nancy Scott, Nkosinathi Ngcobo, Brooke Nichols, Lufuno Malala, Musa Manganye, Sydney Rosen

**Affiliations:** 1 Health Economics and Epidemiology Research Office, Faculty of Health Sciences, University of the Witwatersrand, Johannesburg, South Africa; 2 Department of Global Health, Boston University School of Public Health, Boston, MA USA; 3 Department of Global Health, Amsterdam Institute for Global Health and Development, Amsterdam UMC, University of Amsterdam, Amsterdam, The Netherlands; 4 National Department of Health, Pretoria, South Africa

**Keywords:** HIV, Antiretroviral therapy, Naïve, Treatment interruption, Re-engagement, Metabolite testing, South Africa, Re-initiation, Experience, Interruption, Indicators

## Abstract

**Background:**

The era of universal treatment for HIV has seen high rates of disengagement from antiretroviral therapy (ART) programs and re-engagement after interruptions, with modeled estimates of non-naïve initiators > 50% in many places. Most re-engagers are reluctant to admit prior antiretroviral exposure, and non-self-reported data on proportions reinitiating are scarce. We conducted a sequential, mixed-methods study to explore the proportion of people who present for initiation with evidence of prior ART use and understand why many are reluctant to admit prior exposure in South Africa.

**Methods:**

We enrolled a sequential sample of adults presenting to initiate ART or re-initiate ART after an interruption > 3 months and collected (1) self-reported previous treatment experience; (2) electronic medical record (EMR) evidence of prior ART clinic visits; (3) baseline blood tests for metabolites of tenofovir diphosphate; and (4) laboratory records indicating prior ART-related tests. Interviews were conducted with clients who self-reported no prior ART use but had evidence of metabolites.

**Results:**

Among 89 enrolled participants (median age 32.5, 62% female), 21 (24%) self-reported previously taking ART > 3 months prior to enrolment. An additional 19 (21%) who did not self-report prior exposure had EMR or laboratory evidence of prior ART use, for a total of 40 (45%) clients with known prior treatment exposure at initiation. Sensitivity of self-report was 40% (95% CI: 25–57%), EMR 43% (27–59%), metabolite testing 45% (29–62%), and laboratory records 73% (56–87%). Interviewees (*n* = 11) reported opting to present as naïve because they perceived that disclosure of prior disengagement would cause delays accessing treatment, require additional documentation, and elicit negative responses from healthcare workers. Study limitations included short duration of metabolite detectability, inability to link individuals within the EMR to discern ART experience at other facilities, and lack of baseline viral load testing.

**Conclusions:**

At least 45% of clients initiating ART in South Africa have prior treatment experience, but only a third of re-initiators voluntarily reveal this. Laboratory records yielded the most accurate results for ascertaining prior treatment exposure. As numbers re-engaging in HIV care after a treatment interruption increase, understanding reluctance to self-report ART experience and exploring opportunities to overcome barriers are critical for preventing repeated interruptions.

**Registration:**

The protocol was registered on July 12, 2022 on clinicaltrials.gov (NCT05454839).

**Supplementary Information:**

The online version contains supplementary material available at 10.1186/s12879-025-11340-4.

## Introduction

As rates of HIV treatment with antiretroviral therapy (ART) coverage approach the global target of 95% of HIV-positive individuals who are aware of their status [1], national HIV programs in sub-Saharan Africa are experiencing very high rates of both disengagement from treatment and re-engagement after interruptions [2, 3]. As a result, a large proportion of clients who present at healthcare facilities for treatment initiation are not ART-naïve–they have prior ART exposure and are instead re-starting treatment after a short or long interruption. We recently conducted a systematic review of studies reporting re-initiation proportions and found estimates ranging from a low of 2% of initiators to a high of 53% [4]. Since then, researchers in South Africa’s Western Cape Province have reported re-initiation rates as high as 69% [3].

The take-home message of our review [4] was that data on the proportion of clients who are re-initiators are scarce. The review found only 11 primary data estimates for all of sub-Saharan Africa. The review identified four indicators of prior exposure. Many of the estimates were based on 1) client self-report, and others on 2) baseline (presentation for ART initiation) viral load test results showing existing viral suppression. Others located 3) earlier viral load test results in clients’ clinic or laboratory records, indicating a previous period of treatment. Finally, some studies utilized laboratory testing to seek 4) ARV metabolites in blood, hair, or urine samples. None of these indicators is currently regarded as an independent “gold standard” for identifying prior ART exposure, presumably because each is constrained in some important way. Self-report, which produces the lowest estimates, suffers from clients’ reluctance to reveal previous disengagement from care. Both viral load suppression at baseline and metabolite tests capture only recent interrupters, who are still suppressed from recent ART use or retain metabolites of antiretroviral medications in their blood, both short-term phenomena. Medical records, which have the potential to reveal much earlier ART use, often cannot be located, cannot be linked to the same individual’s clinic record, or are incomplete [5]. Adding to the difficulty in interpreting results is that many of the studies included in the review that did not rely on self-report explicitly excluded anyone who did self-report prior exposure.

Clients who present for treatment initiation with prior ART exposure have, by definition, disengaged from or interrupted treatment in the past. If the reason for the prior interruption has not been addressed, these clients may face greater or different obstacles to long-term retention in care than do naïve patients. Thus, understanding the accuracy of available indicators, describing the proportions and characteristics of initiators who are non-naïve, and being able to match re-initiators with suitable interventions are important for improving treatment program outcomes. To start to build a reliable evidence base on this topic for sub-Saharan Africa, we conducted an exploratory, mixed-methods sub-study that collected multiple indicators of prior use for a sample of ART initiators in three clinics in South Africa.

## Methods

For this study we employed a quantitative dominant, sequential mixed-methods design [6], where the primary function of the qualitative data was for expansion (i.e., explaining the results of the quantitative survey [7]). Clients were enrolled between September 2023 and October 2023. We first asked patients if they had prior ART exposure, then took a dried blood specimen (DBS) to test for the presence of ART metabolites and searched for medical and laboratory record evidence of prior exposure. We then identified discordant responses (i.e., those who self-reported no previous exposure but had metabolites present in their DBS) and conducted qualitative interviews to elicit nuances and stories behind the discordant results.

### Study sites and population

The study sequentially enrolled adults (≥ 18) who reported either newly initiating ART or re-initiating ART after an interruption of more than 90 days at one of three public sector primary healthcare facilities in South Africa, one each in Mpumalanga, KwaZulu-Natal, and Gauteng provinces. The study sites are a subset of facilities participating in the AMBIT SENTINEL study described elsewhere [8]. Participants enrolled in this study were also enrolled in the PREFER survey [9], a questionnaire-based study of preferences for services in a patient’s first six months on antiretroviral therapy for HIV, conducted at the same facilities as the SENTINEL study.

For this sub-study of prior ART experience, a sequential subsample of PREFER participants who self-reported that they were initiating ART for the first time or re-initiating ART after an interruption of at least 90 days were asked for additional consent to create a DBS from their routinely collected blood samples and to have the DBS tested for the presence of ARV metabolites, as described below. Most participants who consented to DBS creation completed the full PREFER survey, although a small number were administered an abbreviated instrument that asked only about prior treatment experience, not the full set of preferences and other questions. Eligibility for the sub-study was confirmed using a survey screening form. PREFER participants who consented and completed study procedures received a voucher equivalent to USD10 as a token of thanks for their time and participation.

Sample size for the sub-study was based on time and resource availability, rather than any expectation of findings. Delays due to ethics approvals and study site willingness to create DBS samples resulted in a smaller sample than originally hoped [9], but enough samples were collected to provide exploratory results.

### Data collection

Data were collected from five sources, as described in Table 1.

**Table 1:** Sources of data and corresponding outcome measures for the sub-study

Source	Description	Outcome measured	Strengths and weaknesses of source
Survey	The PREFER questionnaire was administered to participants by a trained research assistant upon presentation for ART initiation or re-initiation and provision of written consent. PREFER asked questions about prior experiences of HIV care and treatment, barriers to retention in care, and clients’ preferences and expectations for receiving care. The consent form for PREFER allowed access to participants’ medical records and the blood specimens routinely collected by clinic staff at treatment initiation.	“Self-reported ART use” as reported during the survey. Responses included (1) No previous reported ART use; or (2) Previous ART use, but no ART use in the 90 days prior to ART initiation. (Participants with prior ART use in the preceding 90 days were deemed ineligible for this substudy at screening.)	Strengths: No time limitation–self-report should capture any ART use from first exposure. Weaknesses: Disclosure is rare; many ART clients choose not to reveal prior ART experience.
Blood samples for metabolite testing	Nurses at the study sites were trained to prepare dried blood specimen (DBS) samples from the blood specimens collected as part of routine care at ART initiation. All DBS samples were created on the same day as the participant’s venous blood sample collection as part of routine care at ART initiation [10] and stored in a clinic freezer prior to shipping to the analyzing laboratory. Specimens were shipped by air freight on dried ice with temperature monitoring to the Division of Clinical Pharmacology at the University of Cape Town, South Africa. Mass spectrometry was performed to measure for metabolites of tenofovir diphosphate (TDF), which are typically detectable for approximately 90 days, allowing detection of prior use for up to 3 months [11]. Because patients who self-reported any ART use ≤ 90 days prior to study screening were excluded from the study, all metabolite tests would be negative if self-disclosure were accurate. Further details of DBS collection are outlined in Supplementary file 1.	We created a dummy variable labeled “evidence of recent ART use by DBS specimen”, where any detectable TDF was deemed positive. Those with evidence were classified as having levels consistent with regularly taking 4–6 doses per week, 2–3 doses per week, or < 2 doses per week.	Strengths: Quantified concentration data from biological sample. Weaknesses: Short term; metabolites become undetectable after approximately 90 days, meaning that ART use > 90 days prior to presentation would not be detectable.
Electronic medical records	South Africa’s electronic medical record (EMR) for HIV treatment, TIER.Net [5], was accessed for study patients to search for prior HIV treatment interactions with the healthcare system, including clinical consultations and prescriptions. We note that this data source is expected to be quite incomplete over time, as it does not use a consistent identification number to distinguish individual patients. A patient presenting at a different facility, or even at the same facility after an interruption, may be assigned a new TIER.Net record number, effectively concealing any previous receipt of services.	Any entry in an individual’s EMR record since the earliest date in the dataset (2013) indicating ART initiation, monitoring, or medication prescribing or dispensing was designated as “evidence of previous ART use by EMR.”	Strengths: No time limitation. Weaknesses: No unique patient identifier; misses prior initiations of the same client if at a different healthcare facility.
Laboratory records	In South Africa, samples collected for laboratory testing by the National Health Laboratory Service (NHLS) are identified with a unique specimen number (barcode) at the collection facility. We recorded these numbers for the routine blood samples collected on day of ART initiation and reviewed results in the NHLS online portal to confirm if participants had any evidence of prior ART use, such as any prior viral load test results.	“Evidence of previous ART use by lab,” defined as having had one or more viral load tests in the past.	Strengths: No time limitation; can capture prior treatment experience at other facilities; more sensitive than EMR data when using date of birth and name to search. Weaknesses: Viral load testing for monitoring purposes is typically only done after six months in care, therefore underestimating prior exposure for those who initiated care but did not remain in care long enough to qualify for viral load testing.
Qualitative interviews	A subset of participants who self-reported no prior ART use but were found to have ARV metabolites in their blood samples were contacted for telephonic interviews to understand their reasons for not disclosing prior exposure [12]. We first asked why we may have observed the discordant results. Then, guided by the socio-ecologic model [13, 14], we prompted with a series of questions about individual, interpersonal, facility, and community factors that may have influenced their response. These questions included how they felt on the day of the interview, what their circumstances were, what staff could have done to make it easier to disclose, and why they think others may not disclose prior treatment history. We also asked about pre- and post-exposure prophylaxis and, if female, PMTCT* use, as these could have explained presence of metabolites. All participants in these interviews provided written informed consent to be contacted. Interviews lasted approximately 15 min, were audio recorded, translated into English, and transcribed verbatim for analysis. (Interview guide is included as Supplementary file 2.)	Qualitative findings from interviews, key emerging themes.	Strengths: Contextual understanding—the interviews provided in-depth insights into why participants opted to present as naïve prior to their exposure to antiretroviral therapy. Weaknesses: Due to the small, non-random sample used in this qualitative subset, the findings from these interviews may not be broadly generalizable to larger populations.

*Prevention of Mother-to-Child Transmission

Another potential indicator of prior ART exposure is viral suppression at baseline (initiation). However, we were not able to generate baseline viral load results specifically for this study. Routine baseline viral load testing is not recommended in South African guidelines, and only a small number of participants in the sub-study did receive viral load tests from the clinic when they presented for initiation, according to their EMRs. This was done at the discretion of clinic staff, most likely because the client reported previous inconsistent use or the clinic staff suspected prior, recent ART use (i.e., a re-initiator presenting as naïve). For the sake of completeness, we report results of these tests for participants who had them. Since the small number of participants who did receive baseline viral load tests is almost certainly not representative of patients initiating or re-initiating ART, we did not include these data in the main analysis.

### Statistical analysis

Outcomes measured for each data source are shown in Table 1. We used these to create a variable to measure concordance between self-report and all other indicators of prior exposure. Those who self-reported no prior ART use and had no other positive indicators were designated “concordant-naïve.” Those who reported prior ART use but not in the past 90 days and had NHLS and/or EMR data that supported their timeline were labeled “concordant-experienced.” Finally, participants who self-reported no previous ART use but had evidence of prior ART experience by any other indicator were designated “discordant-experienced.”

We first summarized cohort and participant characteristics at ART initiation. Next, we arranged participants based on the three categories of concordance previously defined above and reported aggregate results to illustrate the range and frequency of each type of discordance. Then, we combined all five measures of prior experience into a singular measure as a proxy for a “gold standard” indicator of prior ART use to quantify the proportions that each individual indicator could potentially contribute to identification of prior ART use with available data sources. We estimated the sensitivity and negative predictive value of each of the individual measures (though, as noted, no true “gold standard” exists against which to estimate these metrics). Because any singular positive indicator would deem a participant as treatment-experienced, false positives were not present. We therefore did not report specificity or positive predictive values, as these would equal 100% across all measures. Finally, we investigated factors associated with prior treatment exposure and reported relative risks with 95% confidence intervals (CI).

Interview transcripts were imported into NVivo. Interviews were read through and each question was coded, line by line, using a combination inductive-deductive approach, and a content analysis was conducted. Key emerging themes for each question were summarized and supported by illustrative quotes which were lightly edited for clarity. Results are presented as integrated throughout the narrative using the contiguous approach [12].

### Ethics

The study protocol was reviewed and approved by the Human Research Ethics Committee of the University of Witwatersrand (M220440), the Boston University IRB (H-42726), and by relevant Provincial Health Research Committees through the National Health Research Database in South Africa (KwaZulu-Natal: KZ_202207_014, Mpumalanga: MP_202207_004, Gauteng: GP_202207_049). The protocol is also registered on clinicaltrials.gov (NCT05454839). All participants provided written informed consent for all data collection. Tissue sample assays were performed in accordance with relevant guidelines and regulations. All study procedures complied in full with the Declaration of Helsinki.

## Results

### Study sample

DBS samples were collected for 89 PREFER participants (median age 32.5, years, 62% female) (Table 2), of a total of 158 PREFER survey participants who met eligibility criteria for this sub-study. Of the 89 enrolled in the sub-study between 29 August and 30 October 2023, 80 completed the full PREFER survey, while 9 completed an abbreviated survey. There were no important differences in terms of demographics, level of education, employment, or whether they reported initiating ART for the first time or were returning to care after a period of more than 90 days between the sample enrolled in the sub-study and the full eligible population. Table 2 reports demographic and socioeconomic characteristics of the 80 participants who did complete the full survey.

**Table 2:** Characteristics of study participants who completed full PREFER questionnaire

Characteristic	*N* (%)
N	80
District*	
West Rand, Gauteng Province	21 (24%)
Ehlanzeni, Mpumalanga Province	28 (31%)
King Cetshwayo, KwaZulu Natal Province	40 (45%)
Gender (% female)*	55 (62%)
Age*	
Median (Q1,Q3)	33 (27,38)
18–24 years	7 (8%)
25–49 years	73 (82%)
≥ 50 years	9 (10%)
Marital status	
I have a primary partner or spouse who I live with	25 (31%)
I have a primary partner or spouse but we do not live together	41 (51%)
I do not have a primary partner or spouse at this time	14 (18%)
How many adults and children are living in your household?	
One person	9 (11%)
2–3 people adults	28 (35%)
≥ 4 people	43 (54%)
How many other people in your household have HIV, to your knowledge?	
None	55 (69%)
One	21 (26%)
2 or more	4 (5%)
Do you think of the house you currently live in as your main house?	
Yes	60 (75%)
No, my main house is somewhere else in South Africa	14 (18%)
No, my main house is in another country	6 (8%)
Number of years living in this area	
< 1 year	14 (18%)
1–4 years	20 (25%)
5–9 years	46 (58%)
≥10 years	0
Highest level of education	
Primary or less	35 (44%)
Secondary	33 (41%)
Post-secondary	12 (15%)
What is your primary occupation?	
Formal employment	30 (38%)
Informal employment	9 (11%)
Unemployed	39 (49%)
Student/trainee	2 (3%)
Do you have electricity in your house?	
No	9 (11%)
Yes	71 (89%)
Do you have access to piped water?	
No	3 (4%)
Yes– to house	44 (55%)
Yes– community tap/pipe	33 (41%)
How often do you or the people in your household go without food?	
Never	60 (75%)
Seldom	6 (8%)
Sometimes	13 (16%)
Often	1 (1%)
If a person in your household became ill and 100 rands (approximately $6) was needed for treatment, how difficult would it be for you to find this money?	
Difficult	43 (54%)
Easy	37 (46%)

*For the full sub-study sample of 89 participants; remaining data are only for n=80 who completed the full PREFER questionnaire

### Indicators of prior exposure

Aggregated results of prior ART exposure for the cohort are shown in Table 3, with details for each participant and indicator provided in Supplementary Table 2, ordered by concordance group (last column) as defined in the methods above. (In the tables, “**X**” designates no evidence of prior exposure and **√** designates evidence of the specified exposure). As shown in Tables 3 and 40/89 participants (45%) had evidence of prior exposure to ART, while 49 (55%) did not.

**Table 3:** Exposure to prior ART, by indicator

Concordance	Indicator				Total (*n*)	Sex (*n*, %)		Age, years (*n*, %)			
	Self	EMR	Lab	Metabolite		Male	Female	< 25	25–34	35–44	≥ 45
Concordant, naïve	No	No	No	No	49	20 (41)	29 (59)	3 (6)	24 (49)	16 (33)	6 (12)
Concordant, experienced	Yes	Yes	Yes	Yes	4	1 (25)	3 (75)			3 (75)	1 (25)
Discordant	Yes	Yes	Yes	No	14	7 (50)	7 (50)		8 (57)	5 (36)	1 (7)
	Yes	Yes	No	No	2	2 (100%)			2 (100)		
	Yes	No	Yes	No	1		1 (100)		1 (100)		
	No	Yes	No	Yes	1	1 (100)			1 (100)		
	No	Yes	No	No	1		1 (100)		1 (100)		
	No	No	Yes	Yes	4	3 (75)	1 (25)		2 (50)	2 (50)	
	No	No	Yes	No	6	1 (17)	5 (83)		5 (83)		1 (17)
	No	No	No	Yes	7	2 (29)	5 (71)	2 (29)	2 (29)	2 (29)	1 (14)
Total concordant					53 (60)	21 (40)	32 (60)	3 (6)	24 (45)	19 (36)	7 (13)
Total discordant					36 (40)	16 (44)	20 (56)	2 (6)	22 (61)	9 (25)	3 (8)
Total prior exposure					40 (45)	17 (43)	23 (57)	2(5)	22 (55)	12 (30)	4 (10)

Overall, 55% of participants (*n* = 49) were concordant-naïve across all indicators and 4% concordant-experienced (*n* = 4) with evidence of prior ART use across all indicators. 40% (*n* = 36) were discordant, with at least one but not all indicators showing prior ART use. Among the discordant, half (*n* = 19, 53% of discordant category) self-reported no prior exposure. Clients with concordant results were similar to those with discordant results in terms of proportion female (60% vs. 56%, respectively) and median age at enrolment (34 years vs. 31 years, respectively). Sites in Gauteng (52%) and Mpumalanga (50%) had higher proportions of discordant results than did those in KwaZulu Natal (28%).

Supplementary Table 2 also shows baseline viral loads conducted for 10 participants who received them. All baseline viral loads performed by the clinics were unsuppressed (i.e., did not indicate recent, previous ART exposure). As noted above, these baseline viral load results were unlikely to be representative of the population of interest and were not considered in this analysis.

In Table 4, results for each indicator are aggregated to summarize study outcomes for both the full study sample and the subsample who self-reported no prior exposure. For indicators that are not time-limited, as shown in Table 1 (EMRs and lab records), we also report the proportion of those with record of prior ART use who also indicated prior use on the PREFER survey.

**Table 4:** Proportions showing prior ART exposure, by indicator (*n* = 89)

Indicator	Full sample *N* (%)
Self-report	
No prior treatment experience	73 (82%)
Prior treatment experience but no ART in past 90 days	16 (18%)
Metabolite testing	
No TDF detected	71 (80%)
TDF detected	18 (20%)
If detected, TDF level	
< 2 doses/week	7 (8%)
2–3 doses/week	8 (9%)
4–6 doses/week	3 (3%)
Electronic Medical Record	
No evidence of prior ART	72 (80%)
Evidence of ART > 90 days before study enrollment	17 (20%)
If evidence of ART > 90 days before study enrollment, self-reported previous ART use on survey	15 (88%)
Days between last evidence of ART care and current initiation (median, IQR)	787 (333,1290)
Days since first ART initiation and last evidence of ART care prior to current initiation (median, IQR)	456 (48,1260)
Laboratory records	
No record of earlier viral load test (indication of prior ART use)	70 (79%)
Record of viral load test in past, indicating ART use ≥ 90 days before study enrollment	19 (21%)
If record of viral load test in past, self-reported previous ART use on survey	10 (53%)
Composite result	
Any indicator of prior ART use (experienced)	40 (45%)
No indicator of prior ART use (naïve)	49 (55%)

Most participants self-reported no prior ART experience at all (*n* = 73, 82%). Sixteen (18%) self-reported previously taking ART but with a current treatment interruption of more than three months prior to presenting for ART re-initiation on the day of study enrollment.

Metabolite test results indicated that 18 (20%) participants had detectable TDF metabolites in their blood specimens. Among these 18, 3 (17%) had a level consistent with regularly taking 4–6 doses per week, 8 (44%) 2–3 doses per week, and 7 (38%) < 2 doses per week. Of the 18 with detectable TDF metabolites, only four reported ever previously being exposed to ART, and all stated that the prior exposure was > 90 days before the samples were taken. EMR and laboratory records both indicated that about one fifth of participants had evidence of previous ART; 88% of those with EMRs and 53% of those with lab evidence disclosed this prior use when responding to the PREFER survey. In the EMR, the median time since last evidence of ART treatment–the duration of the interruption as documented in the EMR–was just over two years (787 days). The median time in care between first ART initiation and last evidence of ART care–time on ART prior to interruption–was roughly one and a quarter years (456 days).

### Accuracy of indicators

To compare the accuracy of each indicator in identifying prior ART exposure, we considered the composite measure of treatment experience reported in Table 4, using all five potential indicators of prior exposure, as our gold standard to calculate the sensitivity and negative predictive value (NPV) of each method (Table 5). Evidence of prior treatment in laboratory records was the most sensitive (73%) and had the highest negative predictive value (82%), while self-report fared the worst (sensitivity 40%, NPV 67%). Metabolite testing and EMR evidence had similar sensitivities and NPV. Combining indicators had mixed results for increasing accuracy due to the varying degree of overlap between positive indicators. The sensitivity of self-report plus laboratory record was markedly higher than self-report alone (75% vs. 40%) but was not a substantial improvement over the laboratory record on its own (73%). Of all combinations, laboratory record of prior VL plus TDF metabolite was the most accurate, with a sensitivity of 95% and NPV of 96%.

**Table 5:** Sensitivity and negative predictive value of measures in detecting prior treatment exposure

Method	Sensitivity (95% CI)	Negative predictive value (95% CI)
Self-report	40% (25–57%)	67% (61–82%)
Laboratory record of prior VL	73% (56–85%)	82% (73–88%)
EMR prior evidence	43% (27–59%)	68% (62–74%)
TDF metabolite	45% (29–62%)	69% (63–75%)
Self-report + Laboratory record of prior VL	75% (59–87%)	83% (74–89%)
Self-report + EMR prior evidence	45% (29–62%)	69% (63–75%)
Self-report + EMR prior evidence + Laboratory record of prior VL	93%(80–98%)	94%(85–98%)
Self-report + TDF metabolite	75% (59–87%)	83% (74–89%)
Laboratory record of prior VL + EMR prior evidence	80% (64–91%)	86% (77–92%)
Laboratory record of prior VL + TDF metabolite	95% (83–99%)	96% (86–99%)
EMR prior evidence + TDF metabolite	75% (59–87%)	83% (74–89%)

### Predictors of prior treatment exposure

Associations of client characteristics and prior treatment exposure were investigated (Supplementary Table 1). The proportion of participants who were treatment-experienced was higher for those aged 18–25 years compared to those aged 25–49 years (57% vs. 44%). In addition, the proportion of treatment experience was higher among participants with low literacy levels and those less comfortable with mobile technology compared to those who reported reading well and being very comfortable with mobile phone usage. There was little difference in proportion with prior treatment exposure stratified by gender, education level, or food security.

### Reasons for non-disclosure

Between 30 April and 8 July 2024, qualitative interviews were conducted with 11 of the 18 participants who self-reported no prior ART exposure but had detectable TDF metabolites in their blood specimens. We were unable to contact the remaining seven despite several attempts. Of those contacted, two were from West Rand District, three from Ehlanzeni District, and six from King Cetshwayo District. Six of the 11 were female.

When explaining the discordant results (self-report versus metabolite results), one respondent reported that she had taken pre-exposure prophylaxis (PrEP) within the past few months, though the exact timing was unclear. Despite the presence of metabolites in their DBS, three of the 11 respondents denied known prior use, with one respondent explaining:

*“I have never taken treatment before that day*,* I am sure about that and I was telling truth.” (*Female, West Rand District.)

The remaining seven respondents all intentionally presented as new (naïve) clients on the day of enrollment, but admitted prior use of ART during the interview. Among these seven explanations, three key themes emerged: (1) individual and interpersonal challenges; (2) medication sharing for convenience; and (3) clinic-level interactions.

First, at the individual and interpersonal levels, circumstances and experiences made it physically or emotionally challenging to continue ART. One individual explained he had stopped taking treatment because an injury made it difficult to get to the clinic.

*“I started taking treatment in 2016*,* I got seriously injured and where I was staying at that time it was very difficult for me to go to the clinic for treatment hence I ended stop taking them.”* (Male, Ehlanzeni District.)

Another implied that her partner was not supportive, which she found stressful and logistically difficult, though it is unclear from her interview if she had actually stopped.

*“I was feeling so sad that day and I was scared to disclose a lot during that day [referencing both her partner and ART status]. I think it is stress caused by my partner. He doesn’t want me to take treatment so I end up hiding my treatment in the house.”* (Female, Ehlanzeni District).

Sharing medication for convenience was also a key theme. Several described that it is simpler and easier to share medication than to negotiate time off from work.

*“The issue is I’m someone who is travelling quite a lot*,* so sometimes when my clinic date comes and I am not around*,* I end up sharing treatment with the mother of my children*,* I would appear as someone who has defaulted on treatment at the clinic*,* yet I am using it from the mother of my children. So*,* when I go back to the clinic after a long time appeared as defaulted treatment and I just present as a new patient.”* (Male, King Cetshwayo District).

*"I said no because I have never officially started treatment*,* but I have taken treatment through my partner whenever she was around*,* so I was scared to say it because I have never officially gone and start treatment at the clinic.”* (Male, King Cetshwayo District.)

At the facility level, both logistics and negative provider interaction influenced previous use of ART. Several people described that logistically, it is simpler to start new if a client is returning to care after a lapse or if a client is transferring clinics. Presenting as new patients is preferable to dealing with the consequences of returning or transferring, which they described as including being shouted at by providers and needing transfer paperwork that can be difficult to obtain. One participant implied that the clinic staff found it easier to restart patients as a new patient, than to find their files after an absence.

*“I [had] started treatment in 2017*,* but I stopped taking them for [unknown period of time]*,* because I had an issue with the nurse. I had skipped my next scheduled appointment*,* and when I went back to the clinic the nurse mistreated me so I decided to stop going to the clinic. I was in the queue the entire day and when I was in front of the queue*,* then she told me because I had missed my date I will have to go to the back she will attend me at last and I had asked at work so I was not treated well then*,* I stopped.”* (Female, King Cetshwayo District).

*“It just that the issue of job opportunities I move around a lot*,* so wherever I am at that point in time when I need treatment I go to the nearest clinic where I present myself as a new patient to avoid delays and asked a lot of questions. So*,* in order for me to access treatment easily without being shouted at or asked many questions or required documents such as transfer letters from previous clinics that may lead me not to get treatment*,* I just test then start treatment. So*,* this becomes an easy way to get treatment.”* (Male, King Cetshwayo District).

*“When I relocated to [Clinic X]*,* I went to the clinic and they told me to start afresh as I am like a person who has never taken treatment before*,* they tested me that day and started everything from scratch.”* (Male, King Cetshwayo District.)

When asked about how facility staff could make it easier for clients to share their prior treatment history, emerging themes were almost entirely at the facility-level and included improving privacy, improving tolerance and patience among nurses, increasing counselling and outreach with peer ambassadors, and including clients in the decision-making process, as illustrated below.

*“I think nurses should ask questions about our treatment journeys not tell us*,* including when they have to give us next appointment dates they need to ask us how comfortable are we with such dates are we going to be able to come to the facility and we share our information or experiences with them*,* not being told on what to do and disregards our life situations.”* (Male, Ehlanzeni District.)

When asked why other people in the community may not remember or disclose that they have had prior ART exposure, themes included: (1) forgetting because feeling better; (2) stigma from health facility staff for having defaulted; and (3) that it is simpler to start as a new patient than to present as a re-initiator.

*“If I guess*,* maybe when someone feels better in his or her body may end up forgetting that they have taken treatment before or even remembering when asked because their body is functioning well there are no signs of sickness.”* (Male, King Cetshwayo District.)

*“Maybe [they are] scared just like myself I was scared. Most of the time in clinics they shout at us if you have defaulted or missed appointments. They will shout you so bad making us scared*,* hence they just say they have never taken treatment*,* they know they have but they scared of attitude and treatment they will get that is why they end up not giving correct information or say they never taken treatment before.”* (Male, Ehlanzeni District.)

*“There are a lot of people saying they have never taken treatment when they have. Sometimes us as guys we go to check and after checking they will say we must start treatment and for whatever reason we don’t. Then we go elsewhere to want to start treatment*,* to avoid a long process and a lot of explanation in new facilities people just decide to say they are new on treatment to cut off a lot of questions and challenges to access treatment.”* (Male, King Cetshwayo District.)

## Discussion

In this sequential, mixed-methods study of a sequential sample of adults presenting to initiate or re-initiate HIV treatment in three clinics in South Africa and self-reporting no prior ART use within the past 90 days, 55% were naive, 45% had indicators suggesting prior ART use. Roughly one fifth were found to have ARV metabolites in blood samples indicating recent (within 90-day) use. An additional third had records of prior viral load tests or ART care, evidence that they were likely restarting ART after a short or long interruption. The qualitative interviews of 11 respondents with discordant results suggest a variety of reasons for both interrupting treatment and non-disclosure of prior experience, including deliberately presenting as new to avoid potential stigma from clinic staff and burdensome transfer processes.

The primary strength of this study was the use of multiple data sources to find evidence of prior ART exposure in the same sample. We found a high level of non-concordance among the sources, with only 10% of those who appear to have prior ART experience showing positive indicators across all sources. This is not entirely surprising, as some indicators—self-report and EMR and lab records—represent lifetime experience, while others—metabolite testing (and baseline viral loads, to the extent captured)—reflect only recent use. It does make clear that none of the available indicators alone is sufficient for an accurate estimate. In our study, laboratory records and EMRs contributed the most to identifying prior exposure to ART, while self-report contributed the least.

Metabolite testing and baseline viral load tests, both of which can accurately discern recent ART use, also both pose a number of problems for routine use. Metabolite testing is expensive, can only be carried out in specialized laboratories and, in our analysis, did not perform better than self-report. Baseline viral load tests, which are being introduced into routine care in some settings [15] but are not recommended as part of routine care in South African treatment guidelines, are less expensive but, in the absence of rapid point-of-care technologies, provide information only days or weeks after the initiation visit, by which time many patients have already disengaged from care. Both of these approaches, moreover, only capture ART exposure within the past 1–3 months, before viral rebound or metabolite clearance. In view of this, active review of patients’ clinic records (EMR) and/or laboratory records may be the most practical approach to identifying re-initiators, particularly where such records are available to clinicians in real time at point of care. In South Africa, for example, a counselor or clerk may be able to look up patients’ clinic and lab histories while a patient waits for services and convey any evidence of prior treatment to the nurse before the treatment initiation consultation begins.

This study follows on a systematic review we conducted that found limited data on prior treatment exposure [4]. We found that there was no consistency in defining populations where prior treatment exposure was being reported and varied estimates in prior exposure measured in similar ways. For example, self-reported estimates in the literature range from 2–21% [16–21], which places our estimate of 18% on the high end. When using EMR data, estimates have ranged from 22% in a population that had ART naïveté in the inclusion criteria [22] to 69% in a dataset that considered all initiations and allowed for individuals to be tracked across multiple facilities [3]. Metabolite testing also ranged from 19% when testing only blood specimens for tenofovir and emtricitabine among participants with undetectable viral loads at initiation to 53% when using both blood and hair samples to test for tenofovir, efavirenz and emtricitabine among a study population who all reported being ART naïve [22, 23]. Baseline viral load suppression is another measure reported in the literature with relatively high yield of previous exposure (30–52%) [24, 25]. Among the small number of participants in our study who had a viral load performed as part of their initiation laboratory tests, however, none were suppressed.

A further strength of this study is that in addition to these measures, we also conducted qualitative interviews with participants who had tested positive for ART metabolites after reporting being treatment naïve or not using ART in the preceding 90 days. One interviewee reported using PrEP, which commonly includes tenofovir and can remain detectable in DBS samples for up to 3 months [11]. As PrEP use expands, testing for metabolites of drugs common to PrEP and ART may become less useful. It is concerning that three of the 11 participants we interviewed continued to deny any known prior exposure to ART before the day of enrollment into the study. While it is possible that participants did not remember prior use or were inadvertently exposed through recreational drugs, neither of these explanations seems likely. We speculate that it is more likely that these three individuals simply represent more adamant examples of the seven clients who deliberately presented to the facilities as naïve and determined that it was in their interest not to reveal prior exposure even to researchers.

For those who did reveal to our interviewer that they had deliberately concealed their prior ART use, the quotations included here offer a range of reasons that are consistent with prior research [26, 27] and appear eminently rational from the perspective of the patients. Despite national guidelines that advise clinic staff to be welcoming and encouraging to re-engagers [28–30], there is abundant evidence that facilities and providers deter patients from revealing prior disengagement [26, 27, 31, 32]. Our results indicated that 15 of the 16 clients who reported prior ART use had EMR documentation of HIV care at the same facility. In contrast, of the 24 who reported no prior use but had other indicators of prior treatment, only 2 had EMRs from the originating facility. Given the high rates of mobility in South Africa, it is likely that there would be substantial use of care at other facilities, and suggests clients were even more reluctant to report ART use at other facilities, or that they were less likely to be identified as a returning engager if they sought treatment at a different facility. Previous research suggests that provider disposition is an important driver of patient behavior [33]. Re-engaging patients report being chastised (or worse), sent to the back of the queue, or told to return on another day with an official transfer letter from another facility. All of these actions on the part of providers would lead patients to hide their previous treatment, particularly when initiation procedures for naïve patients have become simpler and faster in recent years [34, 35]. The time required to locate existing files is also an obstacle, compared to opening a new file. It is striking that one respondent reported that clinic staff advised him to present as a new patient, suggesting that staff, too, regard the re-engagement process as burdensome. All of the evidence presented here suggests that South Africa’s “Welcome Back” strategy, designed to encourage re-engagement in care, is not yet fully achieving its desired goals [28, 36].

Our study had a number of limitations, leading us to regard it as exploratory rather than definitive. First, our inclusion criteria required participants to confirm that they were ART naïve or had a treatment interruption of 90 days or more and were informed that we would conduct metabolite testing and search medical records for prior ART exposure. This process may have led some potential participants to self-select out of the study at the time of screening because they were aware of having undisclosed, prior treatment experience, leading us to underestimate the proportion of initiators with positive measures of prior exposure. Second, we could not access EMR data from other clinics participants may have attended previously, making our EMR-based result for prior exposure almost certainly an under-estimate. Transferring from one facility to another, with or without a treatment interruption between sites, is common in South Africa [37, 38] but cannot readily be observed in facility-based records. Third, we did not have baseline viral load test results for most participants. Baseline viral load would add a fifth indicator of recent ARV exposure and would strengthen our overall findings. Fourth, while we are not aware of other studies using multiple data sources in the same sample to compare the sensitivity of different indicators, our sample was small and limits our ability to comment on predictors of prior treatment exposure with confidence. Fifth, our small number of facilities in just three districts makes generalizability to other locations uncertain, such that our results should be regarded as exploratory only. Sixth, there may be a difference between self-reported prior exposure reported to study interviews and that revealed to clinic staff. Finally, our qualitative analysis might have been subject to survey bias and we are uncertain if we reached saturation or predictability, though we did see repeated themes that elicited important nuance. Despite these limitations, our results are consistent with prior studies using single indicators.

## Conclusions

In this exploratory study, we found evidence that nearly half of adults who presented for ART initiation in three clinics in South Africa had previous treatment exposure, although most preferred not to reveal that experience to healthcare providers due to the negative attitude that the healthcare providers portray towards them. To the extent that knowledge of a patient’s prior experience on ART is an important indicator of future outcomes and can guide providers toward effective interventions, these results pose a major challenge to improving the quality of HIV care. These results could further guide and improve the efforts to welcome returning patients back to care. Future work should explore reasons for reluctance to disclose prior ART use, how to identify re-engagers at initiation, interventions needed to support re-engagers, and healthcare providers’ reasons for not prioritizing the re-engagers in care. In the meantime, providers would be justified in regarding prior ART experience as the norm, not the exception, when patients present for ART initiation, and offer information, support, and services accordingly.

## Supplementary Information

Supplementary Material 1: Creation of DBSsamples for metabolite testing.

Supplementary Material 2.

Supplementary Material 3: Participant characteristics by prior exposure status for participants who completed the full PREFER survey.

Supplementary Material 4: Qualitative interview guide.

## Data Availability

Disidentified data generated by the study (questionnaire responses, biological sample results) will be made available in a public data repository within one year of study protocol closure by the responsible ethics committees. Data obtained from participants routinely collected clinic and laboratory records are owned by the South Africa National Department of Health and cannot be shared by the authors.

## Abbreviations

ART: Antiretroviral therapy
DBS: Dried blood specimen
EMR: Electronic medical record
IQR: Interquartile range
NHLS: National Health Laboratory Service (South Africa)
NPV: Negative predictive value
PrEP: Pre-exposure prophylaxis
TDF: Tenofovir diphosphate
VL: Viral load

## References

[1] UNAIDS, The Path That Ends AIDS, Geneva. UNAIDS. 2023. Available from: http://www.wipo.int/amc/en/mediation/rules

[2] Maskew M, Benade M, Huber A, Pascoe S, Sande L, Malala L, et al. Patterns of engagement in care during clients’ first 12 months after HIV treatment initiation in South africa: A retrospective cohort analysis using routinely collected data. PLOS Global Public Health. 2024;4(2):e0002956. 38416789 10.1371/journal.pgph.0002956 PMC10901315

[3] Euvrard J, Timmerman V, Keene CM, Phelanyane F, Heekes A, Rice BD, et al. The cyclical cascade of HIV care: Temporal care engagement trends within a population-wide cohort. PLoS Med. 2024;21(5):e1004407. 38728361 10.1371/journal.pmed.1004407 PMC11125544

[4] Benade M, Maskew M, Juntunen A, Flynn DB, Rosen S. Prior exposure to antiretroviral therapy among adult patients presenting for HIV treatment initiation or reinitiation in sub-Saharan africa: a systematic review. BMJ Open. 2023;13(11):e071283. 10.1136/bmjopen-2022-071283 PMC10660894 37984944

[5] Osler M, Hilderbrand K, Hennessey C, Arendse J, Goemaere E, Ford N et al. A three-tier framework for monitoring antiretroviral therapy in high HIV burden settings. J Int AIDS Soc. 2014;17(1):18908. 10.7448/IAS.17.1.18908. 10.7448/IAS.17.1.18908 PMC4005043 24780511

[6] Morse JM. Approaches to qualitative-quantitative methodological triangulation. Nurs Res. 1991;40(2):120–3. 2003072

[7] Palinkas LA, Aarons GA, Horwitz S, Chamberlain P, Hurlburt M, Landsverk J. Mixed method designs in implementation research. Adm Policy Ment Health. 2010;38(1):44. 10.1007/s10488-010-0314-z PMC3025112 20967495

[8] Pascoe S, Huber A, Mokhele I, Lekodeba N, Ntjikelane V, Sande L, et al. The SENTINEL study of differentiated service delivery models for HIV treatment in malawi, South africa, and zambia: research protocol for a prospective cohort study. BMC Health Serv Res. 2023;23(1):891. 37612720 10.1186/s12913-023-09813-w PMC10463463

[9] Maskew M, Ntjikelane V, Juntunen A, Scott N, Benade M, Sande L, et al. Preferences for services in a patient’s first six months on antiretroviral therapy for HIV in South Africa and Zambia (PREFER): research protocol for a prospective observational cohort study. Gates Open Res. 2024;7:119. 38343769 10.12688/gatesopenres.14682.2 PMC10858017

[10] South African National Department of Health. 2023 ART Clinical Guidelines for the Management of HIV in Adults, Pregnancy and Breastfeeding, Adolescents, Children, Infants and Neonates. Pretoria: NDOH. 2023. Available from: https://knowledgehub.health.gov.za/system/files/elibdownloads/2023-07/National%20ART%20Clinical%20Guideline%20AR%204.5%2020230713%20Version%204%20WEB.pdf

[11] Anderson PL, Liu AY, Castillo-mancilla JR, Gardner EM, Seifert SM, Mchugh C, et al. Intracellular tenofovir-diphosphate and emtricitabine-triphosphate in dried blood spots following directly observed therapy. Antimicrob Agents Chemother. 2018;62(1):1–13. 10.1128/AAC.01710-17 PMC5740314 29038282

[12] Fetters MD, Curry LA, Creswell JW. Achieving integration in mixed methods designs—principles and practices. Health Serv Res. 2013;48(6pt2):2134–56. 24279835 10.1111/1475-6773.12117 PMC4097839

[13] Bronfenbrenner U. Toward an experimental ecology of human development. Am Psychol. 1977;32(7):513–31.

[14] Kilanowski JF. Breadth of the Socio-Ecological model. J Agromed. 2017;22:295–7. 10.1080/1059924X.2017.1358971 28742433

[15] Zambia Ministry of Health. Zambia consolidated guidelines for treatment and prevention of HIV infection. Lusaka: MOH. 2022. Available from: https://www.moh.gov.zm/wp-content/uploads/filebase/Zambia-Consolidated-Guidelines-for-Treatment-and-Prevention-of-HIV-Infection-2020.pdf

[16] Lebelonyane R, Bachanas P, Block L, Ussery F, Abrams W, Roland M, et al. Rapid antiretroviral therapy initiation in the Botswana combination prevention project: a quasi-experimental before and after study. Lancet HIV. 2020;7(8):e545–53. 32763218 10.1016/S2352-3018(20)30187-9 PMC10921550

[17] Rosen S, Maskew M, Larson BA, Brennan AT, Tsikhutsu I, Fox MP, et al. Simplified clinical algorithm for identifying patients eligible for same-day HIV treatment initiation (SLATE): results from an individually randomized trial in South Africa and Kenya. PLoS Med. 2019;16(9):e1002912. 31525187 10.1371/journal.pmed.1002912 PMC6746347

[18] Maskew M, Brennan AT, Venter WDF, Fox MP, Vezi L, Rosen S. Retention in care and viral suppression after same-day ART initiation: One-year outcomes of the SLATE I and II individually randomized clinical trials in South Africa. J Int AIDS Soc. 2021;24:e25825. 34612601 10.1002/jia2.25825 PMC8694178

[19] Dorward J, Drain P, Osman F, Sookrajh Y, Pillay M, Moodley P, et al. Short communication: early antiretroviral therapy is associated with better viral suppression and less HIV drug resistance after implementation of universal treatment in South Africa. AIDS Res Hum Retroviruses. 2020;36:297–9. 31663368 10.1089/aid.2019.0206 PMC7185315

[20] Genet A, Mekonnen Z, Yizengaw E, Mekonnen D. First line antiretroviral treatment failure and associated factors among people living with HIV in Northwest Ethiopia. Afr Health Sci. 2021;21:263–72. 34394306 10.4314/ahs.v21i1.34 PMC8356610

[21] Pry JM, Mwila C, Kapesa H, Mulabe M, Frimpong C, Moono M, et al. Estimating potential silent transfer using baseline viral load measures among people presenting as new to HIV care in lusaka, zambia: A cross-sectional study. BMJ Open. 2023;13:1–8. 10.1136/bmjopen-2022-070384 PMC10231001 37230517

[22] Sithole N, Gunda R, Koole O, Krows M, Schaafsma T, Moshabela M, et al. Undisclosed antiretroviral therapy use at primary health care clinics in rural KwaZulu Natal South africa: A DO-ART trial sub-study. AIDS Behav. 2021;25(11):3695–703. 34097208 10.1007/s10461-021-03319-4 PMC8560667

[23] Mavhandu-Ramarumo LG, Tambe LAM, Matume ND, Katerere D, Bessong PO. Undisclosed exposure to antiretrovirals prior to treatment initiation: an exploratory analysis. South Afr J HIV Med. 2021;22(1):1–10. 10.4102/sajhivmed.v22i1.1200 PMC8063556 33936791

[24] Buju RT, Akilimali PZ, Tran NT, Kamangu EN, Mesia GK, Kayembe JMN et al. Determinants of survival of HIV patients receiving dolutegravir: a prospective cohort study in conflict-affected bunia, Democratic Republic of congo. Int J Environ Res Public Health. 2022;19(16):10220. 10.3390/ijerph191610220. Published 17 Aug 2022. 10.3390/ijerph191610220 PMC9407849 36011850

[25] Kunzweiler CP, Bailey RC, Mehta SD, Okall DO, Obondi E, Djomand G, et al. Factors associated with viral suppression among HIV-positive Kenyan gay and bisexual men who have sex with men. AIDS Care. 2018;30(sup5):S76–88. 30897938 10.1080/09540121.2018.1510109 PMC10669762

[26] Bisnauth MA, Davies N, Monareng S, Buthelezi F, Struthers H, McIntyre J, et al. Why do patients interrupt and return to antiretroviral therapy? Retention in HIV care from the patient’s perspective in johannesburg, South Africa. PLoS ONE. 2021;16:1–15. 10.1371/journal.pone.0256540 PMC8412245 34473742

[27] Rees K, Bisnauth M, O’Connor C, Ramvhulela T, Vali N. Understanding patients reinitiating antiretroviral therapy in two South African districts. South Afr J HIV Med. 2022;23(1):1380. 10.4102/sahivmed.v23il.1380. Published 14 Apr 2022. 10.4102/sajhivmed.v23i1.1380 PMC9082219 35706545

[28] National Department of Health. The South Africa National Welcome Back Campaign Strategy. Pretoria: NDOH. 2021. Available from: https://knowledgehub.health.gov.za/system/files/elibdownloads/2023-04/A5_Welcome%2520Back%2520Campaign_070222_2-%2520FINAL%2520%25281%2529.pdf

[29] Bisnauth M, Rees K. Welcome Back Campaign evaluation: How to encourage re-engagement with ART. Johannesburg: Anova. Available from: https://www.anovahealth.co.za/wp-content/uploads/2019/11/Welcome-Back-Campaign-Evaluation-How-to-encourgae-re-engagement-with-ART2.pdf

[30] South African National Department of Health. Differentiated Models of Care Standard Operating Procedures. Minimum Differentiated Models of Care Package to Support Linkage to Care, Adherence and Retention in Care. Adherence Guidelines for HIV, TB and NCDs Updated April 2023. Pretoria: NDOH, 2023. Available from: https://knowledgehub.health.gov.za/system/files/elibdownloads/2023-05/WEB%20VERSION_South%20African%20National%20Differentiated%20Models%20of%20Care%20SOPs%202023_FINAL18042023.pdf

[31] Bisnauth MA, Davies N, Monareng S, Struthers H, McIntyre JA, Rees K. Exploring healthcare workers’ experiences of managing patients returning to HIV care in johannesburg, South Africa. Glob Health Action. 2022;15(1):2012019. 10.1080/16549716.2021.2012019. 10.1080/16549716.2021.2012019 PMC8765239 35037586

[32] Nhemachena T, Späth C, Arendse KD, Lebelo K, Zokufa N, Cassidy T et al. Between empathy and anger: healthcare workers’ perspectives on patient disengagement from antiretroviral treatment in khayelitsha, South Africa - a qualitative study. BMC Prim Care. 2023;24(1):34. 10.1186/s12875-022-01957-8. 10.1186/s12875-022-01957-8 PMC9878968 36698083

[33] Killian C, West RL, Orrell C, Gifford A, Haberer JE, Halim N et al. Negative clinic experiences as a barrier to care for people with HIV and their impact on patient preferences for intervention support: a qualitative study in Cape Town, South Africa. AIDS Care. 2024:1–10. 10.1080/09540121.2024.2346255. Epub ahead of print. PMID: 38676915. 10.1080/09540121.2024.2346255 38676915

[34] Labhardt ND, Brown JA, Sass N, Ford N, Rosen S. Treatment outcomes after offering same-day initiation of human immunodeficiency virus treatment-how to interpret discrepancies between different studies. Clin Infect Dis. 2023;77:1176–84. 37229594 10.1093/cid/ciad317 PMC10573746

[35] Ford N, Migone C, Calmy A, Kerschberger B, Kanters S, Nsanzimana S, et al. Benefits and risks of rapid initiation of antiretroviral therapy. AIDS. 2018;32:17–23. 29112073 10.1097/QAD.0000000000001671 PMC5732637

[36] Arendse KD, Walker C, Pfaff C, Lebelo K, Cassidy T, Isaakidis P et al. Supporting re-engagement with HIV services after treatment interruption in South africa: a mixed method program evaluation of msf’s welcome service. Sci Rep. 2024;14(1):7317. 10.1038/s41598-024-57774-9. 10.1038/s41598-024-57774-9 PMC10973441 38538754

[37] Espinosa Dice AL, Bengtson AM, Mwenda KM, Colvin CJ, Lurie MN. Quantifying clinic transfers among people living with HIV in the Western cape, South africa: A retrospective Spatial analysis. BMJ Open. 2021;11(12):e055712. 10.1136/bmjopen-2021-055712 PMC8640660 34857581

[38] Leslie HH, Mooney AC, Gilmore HJ, Agnew E, Grignon JS, deKadt J et al. Prevalence, motivation, and outcomes of clinic transfer in a clinical cohort of people living with HIV in North West province, South Africa. BMC Health Serv Res. 2022;22(1):1584. 10.1186/s12913-022-08962-8. 10.1186/s12913-022-08962-8 PMC9791728 36572869

