# Supplementary material for: Prior antiretroviral therapy exposure among clients presenting for HIV treatment initiation in South Africa: an exploratory mixed-methods study using multiple indicators of exposure
Source: BMC Infect Dis. 2025 Jul 26;25:947. doi: 10.1186/s12879-025-11340-4 (PMC12296601; doi:10.1186/s12879-025-11340-4)
Supplement: Supplementary file 2 — Supplementary Material 2. [file 12879_2025_11340_MOESM2_ESM.docx]

Supplementary table 2. Exposure to prior ART for each study participant, by indicator

| **Study #** | **Age range** | **Province** | **Self** | **EMR** | **Lab** | **Metabolite** | **Baseline VL** | **Naïve?** | **Concordance** |
| --- | --- | --- | --- | --- | --- | --- | --- | --- | --- |
| 1 | 35-44 | Gauteng | **X** | **X** | **X** | **X** | **NA** | Yes | Yes, naive |
| 2 | 25-34 | Gauteng | **X** | **X** | **X** | **X** | **NA** | Yes | Yes, naive |
| 3 | 35-44 | Gauteng | **X** | **X** | **X** | **X** | **NA** | Yes | Yes, naive |
| 4 | ≥45 | Gauteng | **X** | **X** | **X** | **X** | **NA** | Yes | Yes, naive |
| 5 | 25-34 | Gauteng | **X** | **X** | **X** | **X** | **NA** | Yes | Yes, naive |
| 6 | 25-34 | Gauteng | **X** | **X** | **X** | **X** | **NA** | Yes | Yes, naive |
| 7 | 35-44 | Gauteng | **X** | **X** | **X** | **X** | **NA** | Yes | Yes, naive |
| 8 | <25 | Gauteng | **X** | **X** | **X** | **X** | **NA** | Yes | Yes, naive |
| 9 | 25-34 | Gauteng | **X** | **X** | **X** | **X** | **NA** | Yes | Yes, naive |
| 10 | 25-34 | Gauteng | **X** | **X** | **X** | **X** | **NA** | Yes | Yes, naive |
| 11 | 25-34 | Mpumalanga | **X** | **X** | **X** | **X** | **NA** | Yes | Yes, naive |
| 12 | 25-34 | Mpumalanga | **X** | **X** | **X** | **X** | **NA** | Yes | Yes, naive |
| 13 | 25-34 | Mpumalanga | **X** | **X** | **X** | **X** | **NA** | Yes | Yes, naive |
| 14 | 35-44 | Mpumalanga | **X** | **X** | **X** | **X** | **NA** | Yes | Yes, naive |
| 15 | 25-34 | Mpumalanga | **X** | **X** | **X** | **X** | **NA** | Yes | Yes, naive |
| 16 | 35-44 | Mpumalanga | **X** | **X** | **X** | **X** | **NA** | Yes | Yes, naive |
| 17 | 35-44 | Mpumalanga | **X** | **X** | **X** | **X** | **NA** | Yes | Yes, naive |
| 18 | ≥45 | Mpumalanga | **X** | **X** | **X** | **X** | **NA** | Yes | Yes, naive |
| 19 | 25-34 | Mpumalanga | **X** | **X** | **X** | **X** | **NA** | Yes | Yes, naive |
| 20 | 25-34 | Mpumalanga | **X** | **X** | **X** | **X** | **NA** | Yes | Yes, naive |
| 21 | ≥45 | Mpumalanga | **X** | **X** | **X** | **X** | **NA** | Yes | Yes, naive |
| 22 | ≥45 | Mpumalanga | **X** | **X** | **X** | **X** | **NA** | Yes | Yes, naive |
| 23 | 35-44 | KwaZulu-Natal | **X** | **X** | **X** | **X** | **NA** | Yes | Yes, naive |
| 24 | ≥45 | KwaZulu-Natal | **X** | **X** | **X** | **X** | **NA** | Yes | Yes, naive |
| 25 | 25-34 | KwaZulu-Natal | **X** | **X** | **X** | **X** | **NA** | Yes | Yes, naive |
| 26 | 25-34 | KwaZulu-Natal | **X** | **X** | **X** | **X** | **NA** | Yes | Yes, naive |
| 27 | <25 | KwaZulu-Natal | **X** | **X** | **X** | **X** | **NA** | Yes | Yes, naive |
| 28 | 35-44 | KwaZulu-Natal | **X** | **X** | **X** | **X** | **NA** | Yes | Yes, naive |
| 29 | 25-34 | KwaZulu-Natal | **X** | **X** | **X** | **X** | **NA** | Yes | Yes, naive |
| 30 | <25 | KwaZulu-Natal | **X** | **X** | **X** | **X** | **NA** | Yes | Yes, naive |
| 31 | 25-34 | KwaZulu-Natal | **X** | **X** | **X** | **X** | **NA** | Yes | Yes, naive |
| 32 | 25-34 | KwaZulu-Natal | **X** | **X** | **X** | **X** | **NA** | Yes | Yes, naive |
| 33 | 35-44 | KwaZulu-Natal | **X** | **X** | **X** | **X** | **NA** | Yes | Yes, naive |
| 34 | 25-34 | KwaZulu-Natal | **X** | **X** | **X** | **X** | **NA** | Yes | Yes, naive |
| 35 | 35-44 | KwaZulu-Natal | **X** | **X** | **X** | **X** | **NA** | Yes | Yes, naive |
| 36 | 25-34 | KwaZulu-Natal | **X** | **X** | **X** | **X** | **NA** | Yes | Yes, naive |
| 37 | 35-44 | KwaZulu-Natal | **X** | **X** | **X** | **X** | **NA** | Yes | Yes, naive |
| 38 | 25-34 | KwaZulu-Natal | **X** | **X** | **X** | **X** | **NA** | Yes | Yes, naive |
| 39 | 25-34 | KwaZulu-Natal | **X** | **X** | **X** | **X** | **NA** | Yes | Yes, naive |
| 40 | 35-44 | KwaZulu-Natal | **X** | **X** | **X** | **X** | **NA** | Yes | Yes, naive |
| 41 | 25-34 | KwaZulu-Natal | **X** | **X** | **X** | **X** | **NA** | Yes | Yes, naive |
| 42 | 35-44 | KwaZulu-Natal | **X** | **X** | **X** | **X** | **NA** | Yes | Yes, naive |
| 43 | 25-34 | KwaZulu-Natal | **X** | **X** | **X** | **X** | **NA** | Yes | Yes, naive |
| 44 | 25-34 | KwaZulu-Natal | **X** | **X** | **X** | **X** | **NA** | Yes | Yes, naive |
| 45 | 25-34 | KwaZulu-Natal | **X** | **X** | **X** | **X** | **NA** | Yes | Yes, naive |
| 46 | 35-44 | KwaZulu-Natal | **X** | **X** | **X** | **X** | **NA** | Yes | Yes, naive |
| 47 | ≥45 | KwaZulu-Natal | **X** | **X** | **X** | **X** | **NA** | Yes | Yes, naive |
| 48 | 35-44 | KwaZulu-Natal | **X** | **X** | **X** | **X** | **NA** | Yes | Yes, naive |
| 49 | 35-44 | KwaZulu-Natal | **X** | **X** | **X** | **X** | **NA** | Yes | Yes, naive |
| 50 | 35-44 | Mpumalanga | **√** | **√** | **√** | **√** | **NA** | No | Yes, experienced |
| 51 | 35-44 | Mpumalanga | **√** | **√** | **√** | **√** | **NA** | No | Yes, experienced |
| 52 | ≥45 | KwaZulu-Natal | **√** | **√** | **√** | **√** | **NA** | No | Yes, experienced |
| 53 | 35-44 | KwaZulu-Natal | **√** | **√** | **√** | **√** | **NA** | No | Yes, experienced |
| 54 | 25-34 | Gauteng | **√** | **X** | **√** | **X** | **NA** | No | Discordant |
| 55 | 35-44 | Gauteng | **√** | **√** | **√** | **X** | **NA** | No | Discordant |
| 56 | 25-34 | Gauteng | **√** | **√** | **√** | **X** | **NA** | No | Discordant |
| 57 | ≥45 | Mpumalanga | **√** | **√** | **√** | **X** | **X** | No | Discordant |
| 58 | 25-34 | Mpumalanga | **√** | **√** | **√** | **X** | **X** | No | Discordant |
| 59 | 25-34 | Mpumalanga | **√** | **√** | **√** | **X** | **X** | No | Discordant |
| 60 | 25-34 | Mpumalanga | **√** | **√** | **√** | **X** | **X** | No | Discordant |
| 61 | 25-34 | Mpumalanga | **√** | **√** | **√** | **X** | **X** | No | Discordant |
| 62 | 35-44 | Mpumalanga | **√** | **√** | **√** | **X** | **NA** | No | Discordant |
| 63 | 25-34 | KwaZulu-Natal | **√** | **√** | **√** | **X** | **NA** | No | Discordant |
| 64 | 25-34 | KwaZulu-Natal | **√** | **√** | **X** | **X** | **NA** | No | Discordant |
| 65 | 35-44 | KwaZulu-Natal | **√** | **√** | **√** | **X** | **NA** | No | Discordant |
| 66 | 25-34 | Gauteng | **X** | **X** | **√** | **X** | **X** | No | Discordant |
| 67 | 35-44 | Gauteng | **X** | **X** | **X** | **√** | **NA** | No | Discordant |
| 68 | 25-34 | Gauteng | **X** | **X** | **√** | **X** | **X** | No | Discordant |
| 69 | 35-44 | Gauteng | **X** | **X** | **X** | **√** | **NA** | No | Discordant |
| 70 | 25-34 | Gauteng | **X** | **X** | **√** | **X** | **NA** | No | Discordant |
| 71 | 25-34 | Gauteng | **X** | **X** | **√** | **√** | **NA** | No | Discordant |
| 72 | <25 | Gauteng | **X** | **X** | **X** | **√** | **NA** | No | Discordant |
| 73 | 25-34 | Mpumalanga | **√** | **√** | **√** | **X** | **X** | No | Discordant |
| 74 | ≥45 | Mpumalanga | **X** | **X** | **X** | **√** | **X** | No | Discordant |
| 75 | ≥45 | Mpumalanga | **X** | **X** | **√** | **X** | **NA** | No | Discordant |
| 76 | 25-34 | Mpumalanga | **X** | **X** | **√** | **X** | **NA** | No | Discordant |
| 77 | 35-44 | Mpumalanga | **√** | **√** | **√** | **X** | **X** | No | Discordant |
| 78 | 35-44 | Mpumalanga | **X** | **X** | **√** | **√** | **NA** | No | Discordant |
| 79 | 25-34 | KwaZulu-Natal | **√** | **√** | **√** | **X** | **NA** | No | Discordant |
| 80 | 25-34 | KwaZulu-Natal | **X** | **X** | **X** | **√** | **NA** | No | Discordant |
| 81 | <25 | KwaZulu-Natal | **X** | **X** | **X** | **√** | **NA** | No | Discordant |
| 82 | 25-34 | KwaZulu-Natal | **√** | **√** | **X** | **X** | **NA** | No | Discordant |
| 83 | 25-34 | KwaZulu-Natal | **X** | **√** | **X** | **√** | **NA** | No | Discordant |
| 84 | 25-34 | KwaZulu-Natal | **X** | **X** | **√** | **X** | **NA** | No | Discordant |
| 85 | 35-44 | KwaZulu-Natal | **√** | **√** | **√** | **X** | **NA** | No | Discordant |
| 86 | 25-34 | KwaZulu-Natal | **X** | **X** | **√** | **√** | **NA** | No | Discordant |
| 87 | 25-34 | KwaZulu-Natal | **X** | **√** | **X** | **X** | **NA** | No | Discordant |
| 88 | 25-34 | KwaZulu-Natal | **X** | **X** | **X** | **√** | **NA** | No | Discordant |
| 89 | 35-44 | KwaZulu-Natal | **X** | **X** | **√** | **√** | **NA** | No | Discordant |

“**X**” designates no evidence of prior exposure and **√** designates evidence of the specified exposure
