# Supplementary material for: Prior antiretroviral therapy exposure among clients presenting for HIV treatment initiation in South Africa: an exploratory mixed-methods study using multiple indicators of exposure
Source: BMC Infect Dis. 2025 Jul 26;25:947. doi: 10.1186/s12879-025-11340-4 (PMC12296601; doi:10.1186/s12879-025-11340-4)
Supplement: Supplementary file 3 — Supplementary Material 3: Participant characteristics by prior exposure status for participants who completed the full PREFER survey. [file 12879_2025_11340_MOESM3_ESM.docx]

**Supplementary table 1: Participant characteristics by prior exposure status for participants who completed the full PREFER survey**

| **Characteristics** | **≥1 indicator of**  **prior exposure** | **No prior exposure** | **Relative risk**  **(95% CI)** |
| --- | --- | --- | --- |
| N | 38 | 42 |  |
| District |  |  |  |
| West Rand | 11 (52%) | 10 (48%) | Reference |
| Ehlanzeni | 16 (59%) | 11 (41%) | 0.86 (0.45-1.62) |
| King Cetshwayo | 11 (34%) | 21 (66%) | 1.38 (0.82 – 2.30) |
| Age |  |  |  |
| 18-25 | 4 (57%) | 3 (43%) | Reference |
| 25-49 | 32 (44%) | 41 (56%) | 1.31 (0.54 – 3.16) |
| 50+ | 4 (44%) | 5 (56%) | 1.30 (0.46 – 3.65) |
| \| Gender \| \| --- \| \| Female \| \| Male \| | 24 (48%)  14 (47%) | 26 (52%)  16 (53%) | Reference  1.02 (0.67 – 1.57) |
| Highest level of education |  |  |  |
| Primary or less | 20 (57%) | 15 (43%) | Reference |
| Secondary or more | 18 (40%) | 27 (60%) | 1.40 (0.89 – 2.20) |
| Marital status |  |  |  |
| I have a primary partner/spouse who I live with | 11 (44%) | 14 (56%) | Reference |
| Primary partner/spouse lives elsewhere | 19 (46%) | 22 (54%) | 0.96 (0.61 – 1.50) |
| No current primary partner/spouse | 8 (57%) | 6 (43%) | 0.77 (0.38 – 1.54) |
| How many other people in your household have HIV, to your knowledge? | | | |
| None | 25 (45%) | 30 (55%) | Reference |
| One or more | 13 (52%) | 12 (48%) | 0.88 (0.55 – 1.41) |
| What is your reading level? |  |  |  |
| Read well | 30 (45%) | 37 (55%) | Reference |
| Read somewhat | 8 (62%) | 5 (38%) | 0.70 (0.34 – 1.43) |
| How comfortable are you using a mobile phone or computer for receiving information | | | |
| I am very comfortable | 27 (43%) | 36 (57%) | Reference |
| I am somewhat comfortable | 11 (65%) | 6 (35%) | 0.62 (0.31 – 1.22) |
| What is your primary occupation? |  |  |  |
| Employed | 15 (38%) | 24 (62%) | Reference |
| Unemployed/Trainee | 23 (56%) | 18 (44%) | 0.71 (0.47 – 1.09) |
| Do you have electricity in your house? | | | |
| No | 3 (33%) | 6 (67%) | Reference |
| Yes | 35 (49%) | 36 (51%) | 0.76 (0.45 – 1.27) |
| Do you have access to piped water? | | | |
| No | 2 (67%) | 1 (33%) | 0.59 (0.12 – 2.97) |
| Yes – to house | 19 (43%) | 25 (57%) | Reference |
| Yes – community tap/pipe | 17 (52%) | 16 (48%) | 0.85 (0.55 – 1.32) |
| Do you or the people in your household go without food often, sometimes, seldom? | | | |
| Never/seldom | 31 (47%) | 35 (53%) | Reference |
| Sometimes/Often | 7 (50%) | 7 (50%) | 0.94 (0.53 – 1.67) |
| If a person in your household became ill and 100 Rands was needed for treatment, how difficult would it be for you to get it? | | | |
| Difficult | 23 (52%) | 21 (48%) | Reference |
| Easy | 16 (43%) | 21 (57%) | 1.19 (0.78 – 1.81) |
