# Supplementary material for: Prior antiretroviral therapy exposure among clients presenting for HIV treatment initiation in South Africa: an exploratory mixed-methods study using multiple indicators of exposure
Source: BMC Infect Dis. 2025 Jul 26;25:947. doi: 10.1186/s12879-025-11340-4 (PMC12296601; doi:10.1186/s12879-025-11340-4)
Supplement: Supplementary file 4 — Supplementary Material 4: Qualitative interview guide. [file 12879_2025_11340_MOESM4_ESM.pdf]

PREFER: ART Metabolite Semi-structured interviews

Field	Question	Answer
Form control		
surveyor_id *(required)*	Surveyor ID	[redacted]
specify_surveyor *(required)*	Specify the surveyor	
district *(required)*	District name	
wr_facilities *(required)*	Facility name	
mp_facilities *(required)*	Facility name	
kzn_facilities *(required)*	Facility name	
interviewdate	Date<br>*Today's date*	
sid *(required)*	Survey ID options	1 Barcode<br>2 Enter manually
barcode_scan *(required)*	Scan survey ID	
participant_id *(required)*	Survey ID	
confirm	Step 1: Confirmation<br>*Surveyor: Confirm that you are talking to the participant and not someone else.*	
intro	Step 2: Introduce yourself<br>*Hello, I am .... from the Health Economics and Epidemiology Research office at the University of Witwatersrand Faculty of Health Sciences. A while back, you participated in our PREFER study when you started treatment for HIV. As part of the study, you answered some of our questions and gave permission for us to test your blood and contact you again if we had more questions. We are contacting you again now because we have a few more questions and trying to understand some of the information we collected.*	
Step 3: Screening		
willing	1. Are you willing to talk to us for about 15-20 mins about your past experiences in getting treatment for HIV?	1 Yes<br>0 No
convenienttime	Is now a convenient time to talk	1 Yes<br>0 No
scheduletime	2. Can we please schedule a new and convenient time:	1 Yes<br>0 No
convenientdate	Which date will be convenient for you?	
Step 4: Questionnaire		
gettingart	1. As of today, are you still getting treatment at the same facility where you started treatment when we first interviewed you?	1 Yes<br>0 No
explain	Please explain	
art_inbody	2. When we last spoke, we asked you about previous times that you received treatment for HIV. Our records show that you either had never received treatment or that you had not received treatment in the 3 months before to coming to the clinic that day. The tests we did on your blood however, indicate that you had medications used for the treatment of HIV in your body before you started treatment on that day. We've heard stories that some people sometimes share medication, or take tablets once in a while so don't consider it 'taking medication', or don't want to report having previously taken medication. We also know that some bodies process medication differently. Can you help us to understand this by sharing how that HIV medication could have been in your body when you thought it wasn't?	
takenart_where	3.Thinking back to that day, do you have any idea where you could have taken HIV medication before that day? Remember that even taking one tablet in the prior 3 months would show up in the tests we did. Please explain whatever you can remember.<br>*Probe with: i. Had anyone shared their ART with you or given you some to try? Have you shared your ART with anyone else? Please tell me that story. ii. Had you gotten ART at a different facility? Or a pharmacy using a private GP script? iii. Is the clinic where you initiated when we spoke to you the only place you are getting treatment for HIV from?*	
howyoufelt	4. Can you explain how you felt when we asked you about previously taking medication at that visit?<br>*Probe: were you reluctant/scared to share with us, forgot, didn't think it was relevant? Why?*	
circumstances	5. Were there circumstances in your family, community or the clinic that could make it harder to share information on your treatment use? What would make it more difficult to share this with your health provider?	
makeit_easier	Can you think of ways the facility or health workers could make it easier to share this information?	
notknow	6. Why might other people in your community not know or remember that they had taken HIV medication? Please explain or tell a story if you've heard of someone.	
takenprep	7. Had you ever taken PrEP (Pre-Exposure Prophylaxis) or PEP (post-exposure prophylaxis)	1 Yes<br>0 No
gender *(required)*	Surveyor: Please enter the participant's gender	1 Male<br>2 Female

Field	Question	Answer
pregnant	8. At the time of the visit, were you pregnant or breastfeeding?	0 Neither pregnant nor breastfeeding<br>1 Pregnant<br>2 Breastfeeding
currently	Are you currently pregnant or breastfeeding	0 Neither pregnant nor breastfeeding<br>1 Pregnant<br>2 Breastfeeding
anythingelse	Is there anything else you'd like to share to help us understand how the medication was in your body before you officially started the day we interviewed you?	
closing	Please thank the participant for their time and ask if they have questions about the study.	
sid_2 *(required)*	Survey ID options	1 Barcode<br>2 Enter manually
barcode_scan2 *(required)*	Scan survey ID	
participant_id2 *(required)*	Survey ID	
notesend *(required)*	Surveyor notes	
refusal	STOP. Thank the participant for their time.	
refusaldetails	Indicate that participant was successfully contacted, but did not want to talk (refusal):<br>*State reason for refusal*	
